# Role of Basic Surface Groups of Activated Carbon in Chlordecone and β-Hexachlorocyclohexane Adsorption: A Molecular Modelling Study

**DOI:** 10.3390/molecules26226969

**Published:** 2021-11-18

**Authors:** Kenia Melchor-Rodríguez, Chayan Carmenate-Rodríguez, Anthuan Ferino-Pérez, Sarra Gaspard, Ulises J. Jáuregui-Haza

**Affiliations:** 1 Instituto Superior de Tecnologías y Ciencias Aplicadas, University of Havana, Havana 10400, Cuba; keniamr@instec.cu (K.M.-R.); chayancarmenate@gmail.com (C.C.-R.); anthuanferp@gmail.com (A.F.-P.); 2 Laboratoire COVACHIM M2E, EA 3592, Université des Antilles, 97110 Pointe-à-Pitre, Guadeloupe, France; sarra.gaspard@univ-antilles.fr; 3 Instituto Tecnológico de Santo Domingo (INTEC), Santo Domingo 10602, Dominican Republic

**Keywords:** chlordecone, beta-hexachlorocyclohexane, activated carbon, nitrogen-containing functional groups, computational chemistry

## Abstract

The influence of nitrogen-containing surface groups (SGs) onto activated carbon (AC) over the adsorption of chlordecone (CLD) and β-hexachlorocyclohexane (β-HCH) was characterized by a molecular modelling study, considering pH (single protonated SGs) and hydration effect (up to three water molecules). The interactions of both pollutants with amines and pyridine as basic SGs of AC were studied, applying the multiple minima hypersurface (MMH) methodology and using PM7 semiempirical Hamiltonian. Representative structures from MMH were reoptimized using the M06-2X density functional theory. The quantum theory of atoms in molecules (QTAIM) was used to characterize the interaction types in order understanding the adsorption process. A favorable association of both pesticides with the amines and pyridine SGs onto AC was observed at all pH ranges, both in the absence and presence of water molecules. However, a greater association of both pollutants with the primary amine was found under an acidic pH condition. QTAIM results show that the interactions of CLD and β-HCH with the SGs onto AC are governed by Cl···C interactions of chlorine atoms of both pesticides with the graphitic surface. Electrostatic interactions (H-bonds) were observed when water molecules were added to the systems. A physisorption mechanism is suggested for CLD and β-HCH adsorption on nitrogen-containing SGs of AC.

## 1. Introduction

Chlordecone (CLD, C_10_Cl_10_O), and hexachlorocyclohexane (HCH, C_6_H_6_Cl_6_) chlorinated pesticides were extensively used in Martinique and Guadeloupe until the beginning of the 1990s to prevent the propagation of the banana weevil (Cosmopolite sordidus), which attacks the roots of the banana tree, resulting in the contamination of soil and surface waters [1,2]. The French Ministry of Agriculture withdrew authorization for CLD in 1990, but its use in the French departments continued until September 1993 [3]. Due to its deleterious effects on the environment and organisms, chlordecone has been classified as a persistent organic pollutant (POP) and was banned globally in 2011 by the Stockholm Convention on POP [4]. The persistence of CLD in the environment can be explained by its poor biodegradability [5,6] due to its chemical structure and high steric hindrance. Chlordecone has a high bioaccumulation factor in organisms, is highly lipophilic, persists in the environment and organisms, and has a broad range of toxicities, including neurotoxicity. Sadly, the islands of Martinique and Guadeloupe show the highest prostate cancer diagnosis rates in the world, which could be related to pesticides exposure [3].

Hexachlorocyclohexane (HCH) is a monocyclic, saturated, chlorinated hydrocarbon. Its six chlorine atoms make it stable in the environment [7]. HCH was spread in the form of technical HCH consisting of eight isomers, the water solubility of which varies between 5 (for β-HCH) and 20 mg/L, and vapor tension between 3.54 × 10^−5^ (for β-HCH) and 0.003 Pa at 20 °C [8]. Mainly, four of them are found in technical-grade products, β-HCH (accounting for 5 to 12% of technical HCH) being considered the most recalcitrant [9]. β-HCH is the isomer with the greatest tendency to bioaccumulate and biomagnificate (Figure 2). The predominant formation of significant amounts of β-HCH from other isomers has been demonstrated, resulting in an increment of its stability and a reduced bioremediation rate, causing an increase in its environmental persistence [10]. It has been also established that β-HCH is neurotoxic, hepatotoxic, causes fertility problems, has immunosuppressive effects, and acts as an endocrine disruptor [10,11]. As a result, in 2009, β-HCH was included in the list of persistent organic pollutants (POPs) by the Stockholm Convention [4].

Recently, the utilization of carbon-based materials as a highly efficient adsorbent for pollution remediation has drawn more and more attention due to their versatility and favorable properties, such as high surface area, porosity, and specific chemical properties, which allow for interaction with different chemical compounds [12,13]. However, the surface chemistry of the carbon is also an important factor; this is evident from the constantly increasing number of publications dealing with this topic [13,14,15,16]. Indeed, the efficiency of AC adsorption strongly depends on the specific interactions between adsorbent and adsorbate, which, in turn, mainly depend on their chemical properties [13]. In addition, the adsorption mechanism is also influenced by the solute properties, such as molecular size, solubility, ability for dissociation, and physicochemical properties [17]. However, even though ACs are commonly used in water treatment for removal of inorganic and organic molecules [16,18,19], knowledge of its adsorption mechanism for environmental pollutants is very limited, and for many pollutants, such knowledge does not exist at all.

In this sense, the theoretical modeling can help to understand the adsorption process and may lead to improvements in the AC selection process. In fact, in previous works by our research group, theoretical studies employing the multiple minima hypersurface (MMH) [20,21] computational methodology were undertaken in order to help to understand the influence of acidic AC surface groups (SGs) on CLD [22] and β-HCH [23,24], adsorption. The use of the MMH methodology allows, on one hand, to explore the possible interaction sites of the surface groups of the AC with the water molecules and contaminants, and on the other hand, to calculate the thermodynamic properties of the interacting system. These may provide a first selection criterion for the preparation of AC with suitable surface properties. Later, MMH, high-level quantum calculations based on density functional theory (DFT) [25,26], and a topological exploration of the electron density based on the quantum theory of atoms in molecules (QTAIM) [27,28] approach were successfully used to identify the main interactions types of CLD with acidic SGs (hydroxyl and carboxyl) under different pH and solvation conditions [29,30,31]. These theoretical results are consistent with experimental findings [22], indicating that the AC with the highest content of COOH SGs showed the strongest CLD/SG interaction under slightly acidic and neutral pH conditions, considering Nakanishi’s criteria [32,33].

Previously, molecular modeling studies of chlordecone and metaldehyde interactions with acidic activated carbon surface functional groups have been published using this methodology [24,29,30]. The present methodology was also used to theoretically evaluate the molecular inclusion process between CLD and cyclodextrins [34]. In addition, Jáuregui-Haza et al. [35], studied the guest-host complexes of 1-iodochlordecone and β-1-iodo-pentachlorocyclohexane with cyclodextrins to use them as radiotracers of organochlorine pesticides in polluted water.

Now, it is important to focus the attention on finding the best surface groups and operational conditions that enhance the adsorption process of pesticides into activated carbon, which is the main goal of the present work. To our knowledge, there are not molecular modeling studies of the interaction of CLD and β-HCH with AC basic surface groups [31]. Taking into account the above-mentioned experimental and theoretical results, the present work focuses on evaluating, for the first time, the influence of some of the functionalities of AC basic surface groups, such as nitrogen-containing groups, over the adsorption of CLD and β-HCH, considering pH and solvent effects.

## 2. Materials and Methods

### 2.1. System under Study: Activated Carbon Model

Models that correctly describe ACs’ morphology, topology, and constitution usually do so at a high computational cost. Therefore, the use of small polycycles, such as coronene [24,29,30,36,37,38,39,40], and their oxidized derivatives is very popular for theoretical studies that aim to characterize their interactions with a variety of molecules. The use of such small models saves computational resources and allows either an application of a relatively high level of calculations or a thorough exploration of the interactions present in the system under study [29].

On the other hand, this molecule is considered the simplest molecular structure compatible with the size of the graphitic fragments observed experimentally in activated carbons (>1 nm). For that reason, it was used as a carbon model base (or building unit) when more complex or realistic models of AC are created [41,42]. 

**Figure 1:**
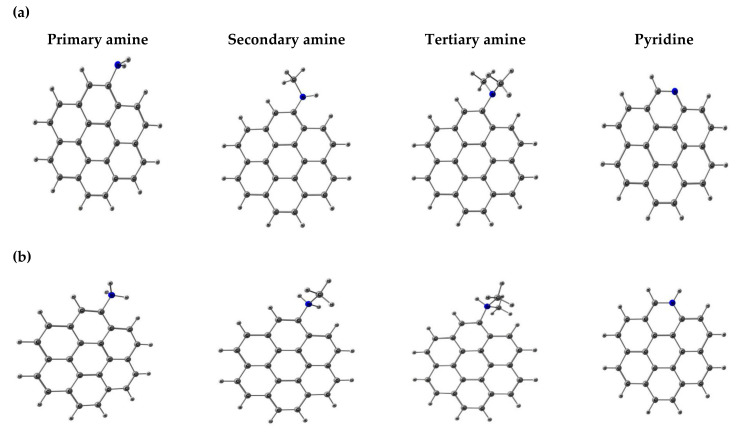
Activated carbon model for the molecular modelling studies: (**a**) nitrogen surface groups and, (**b**) protonate nitrogen surface groups.

Hence, a model of AC made up of a seven-membered-ring graphene sheet (coronene) was selected to study the effect of nitrogen basic surface groups (SGs) during adsorption of CLD and β-HCH. This model neglects the influence of pore shape, defects, pore size, and pore connectivity but instead allows the performance of a large number of calculations with a higher theoretical level in order to achieve a better chemical description of the studied phenomena. This study only considers modifications in the edge of coronene that do not affect the aromaticity of the system. Depicted in Figure 1a,b, the models provide either the aromatic character or graphene structure and the SGs at the edges of AC. In addition, this model was selected to compare the results obtained in the present work with previous works of our research group related to the study of the adsorption process of CLD and β-HCH onto acidic AC [24,29,30].

### 2.2. Basic Surface Groups

Surface functionalities of activated carbons have been traditionally split into two families attending to their acidic or basic character in aqueous solution. There is a general agreement on the type of surface functionalities that account for the acidic character of a carbon material (i.e., carboxyl groups, lactones, phenol, and lactol groups). Nevertheless, this is not the case for basic carbon surfaces, with several models of basic oxygen-containing functionalities being proposed: chromene structures, diketone or quinone groups, and pyrone-like groups [43]. There is no consensus about the strength as bases of these groups and the extent of their contribution to the overall carbon basicity. 

However, the basicity of activated carbon can be associated with: (i) resonating electrons of carbon aromatic rings that attract protons, and (ii) basic surface functionalities (e.g., nitrogen-containing groups) that are capable of binding with protons [44]. Furthermore, the pH variation can enhance the interaction between carbon surfaces and acid molecules by dipole-dipole interaction, H-bonding, and covalent bonding. Nitrogen-containing functionalities can be introduced through either reaction with nitrogen-containing reagents (such as NH_2_, nitric acid, and amines) or activation with nitrogen-containing precursors [44]. As acidic surface groups, these functional groups are concentrated principally on the edges of graphene layers. Possible structures of the nitrogen functionalities include the following: amide, imide, lactame, pyrrolic, and pyridinic groups [44].

**Figure 2:**
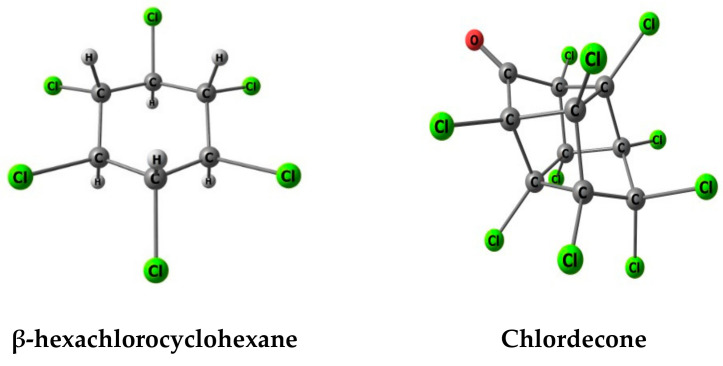
Pesticide computational models.

Accordingly, and with the aim of centering the attention on the influence of AC basic nitrogen-containing surface groups on the adsorption process of CLD and β-HCH, the following SGs were selected for this study: primary amine (-NH_2_), secondary amine (-NHCH_3_), tertiary amine [-N(CH_3_)_2_] (directly attached to an sp2 carbon), and pyridine (-Pyr). Figure 1a, presents the computational models of these nitrogen-containing SGs representing, in the current work, the neutral and basic pH conditions. To consider pH influence (acidic pH), protonated models with -NH_3_^+^, -NH_2_CH_3_^+^, -NH(CH_3_)_2_^+^, and -PyrH^+^ were also studied (Figure 1b). In order to evaluate the solvation process, up to three water molecules were added to the systems. Figure 2 presents the computational models of the pesticides β-HCH and CLD.

### 2.3. Computational Methodology

For the evaluation of the adsorption energies of the guest molecules in carbon-based material models, computational methods have been widely used [15,45,46]. However, the description of interactions between adsorbents and AC surfaces still represents a challenge for the scientific community using current theoretical techniques. 

Concerning that, the multiple minima hypersurface methodology [20,21] was used to explore the configurational space of the CLD and β-HCH interactions with the selected basic nitrogen-containing surface groups onto activated carbon. Afterward, distinctive minima structures were selected that represent the possible types of interaction in the systems under study. Next, the selected distinctive minima structures obtained from MMH were reoptimized using the density functional theory [25,26]. Finally, the quantum theory of atoms in molecules [27,28] was used in order to describe more accurately the nature of the interactions in the systems under study. Previously, molecular modeling studies of chlordecone and metaldehyde interactions with acidic activated carbon-surface functional groups have been published using this methodology [22,27,28]. The present methodology was also used to theoretically evaluate the molecular inclusion process between CLD and cyclodextrins [39]. In addition, Jáuregui-Haza et al. [40] studied the guest-host complexes of 1-iodochlordecone and β-1-iodo-pentachlorocyclohexane with cyclodextrins to use them as radiotracers of organochlorine pesticides in polluted water.

### 2.4. Multiple Minima Hypersurface Methodology

MMH procedure has been successfully employed for evaluating the configurational space of CLD and β-HCH interactions with activated carbon surfaces. Durimel et al. [22,24] and Enriquez-Victorero et al. [23] have published theoretical results on CLD and β-HCH interactions with functional groups using MMH methodology. Generally, MMH methodology is based on semiempirical methods. The semiempirical Hamiltonian PM7 [47] was used in the present work for the optimization of the 700 geometries using the software MOPAC2016 [48]. PM7 is a semiempirical Hamiltonian that improves the description of the H-bond and dispersive interactions with respect to the previous semiempirical methods, except for PM6-D3H4X. Even though the PM6-D3H4X method gives the best results, PM7 yields only slightly inferior results and brings additional improvements in the description of other molecular properties. Therefore, PM7 can be recommended as the most robust semiempirical quantum mechanical method. Taking that into account, the semiempirical Hamiltonian PM7 enhances the description of dispersive interactions and hydrogen bonds compared to preceding semiempirical procedures [47]. 

This methodology begins with the generation of the starting structures. The initial geometries for the water molecules, pollutants, as well as all the basic nitrogen-containing surface groups (protonated and neutral) of the AC model were created using the graphical assistant of the program Chemcraft [49]. Subsequently, each of the structures was optimized separately with the program package MOPAC2016 [48], using the semiempirical Hamiltonian PM7 [47]. 

Then, 700 non-redundant configurations for the GS/CLD/(H_2_O)_n=0–3_ and GS/β-HCH/(H_2_O)_n=0–3_ systems (considering the structure of protonate nitrogen-containing SGs as a function of pH) were randomly generated from the structures of the isolated molecules. Additionally, the coronene/(H_2_O)_n=1-3_ and pesticide/(H_2_O)_n=0–3_ systems were considered in order to be used as reference (Tables S1 and S2, Supplementary Materials). Previous studies of similar systems showed that 700 configurations are sufficient for the association energy of the molecular complexes to converge on a fixed value [23,50]. The hypothesis of a maximum of three water molecules was considered, as previous studies show that for similar systems, this number is sufficient to saturate the surface OH and COOH groups [23,29,30,31,39,51]. Considering that the adsorption of CLD and β-HCH takes place in an aqueous medium, the GS/(H_2_O)_n=0–3_ systems were also studied as reference. To generate the configurations, the program GranadaR2 [44] was used. 

The generated supermolecules were used as input files for the semiempirical program package MOPAC2016 [48], through which the minimum energy structures were obtained after a second optimization. These structures were processed using the Q3 program [21] that employs statistical thermodynamics methods in order to obtain the thermodynamic magnitudes of association of the systems under study and to select the most important non-redundant structures for their description. After such mathematical treatments, the most populated structures were selected according to a Boltzmann distribution of energy states. Although Gibbs’s free energy of association (ΔG_ASSOC_) is the thermodynamic criteria used to define the spontaneity of a process, the association energy (ΔE_ASSOC_) was used in this study. This criterion has been profusely used by previous researchers [22,23,29,30,34,35,39,40] since it is computationally simpler compared to ΔG, and it avoids having to calculate the association entropies. It is defined as $\Delta E_{\mathrm{ASSOC}}=E_{\mathrm{supermolecule}}-E_{\mathrm{ref}}$, where E_supermolecule_ is the energy of the formed molecular complex by the activated carbon model (with and without SGs) and the interacting in the studied system molecules (in this case: CLD, β-HCH, and water molecules) and E_ref_ is the energy of the isolated molecules. A favorable thermodynamic association implies that the supermolecule will be more stable than the isolated molecules, namely a greater absolute value of ΔE will correspond to more energetically favorable associations [29]. Given that, if the molecular energy is negative, then ∆E_ASSOC_ will be negative; thus, a major absolute value of ∆E_ASSOC_ is in correspondence with a more energetically favorable association. In all calculations, the eigenvector-following routine, ‘‘EF’’, for searching minima was used.

### 2.5. Reoptimization by Density Functional Theory

DFT is an alternative ab initio method that has posed itself as one of the most popular and versatile methods available for the study of the electronic structure of innumerable systems. In the present work, DFT-based calculations [25,26], were used to reoptimize the most stable distinctive structures, after MMH calculations, to obtain a geometrical and electronic description of better quality that permits a more rigorous characterization of the nature of interactions present in the systems under study: SG/CLD/(H_2_O)_n=0–3_ and SG/β-HCH/(H_2_O)_n=0–3_. For this reoptimization, a hybrid meta-generalized gradient functional, M06-2X [52,53], with the eople basis 6-31+G(d,p), for all atoms was employed. This combination has been reported to correctly describe the interactions found [53], particularly the van der Waals-type interactions. All calculations were performed with the Gaussian09 software package [54]. In all performed quantum calculations, the geometries and wave-function optimizations were carried out without symmetry constraint for both geometrical and electronic structure. The selected keywords of DFT input files are additionally presented: (1) *Empirical Dispersion = GD3*: to better describe non-covalent interactions; (2) *Opt = (CalcAll, Cartesian)*: optimize the structures, minimizing them with a quadratic convergence (Newton specification) and using Cartesian coordinates (Cartesian specification); (3) *Int = UltraFine*: utilized to increase the numerical accuracy; (4) *NoSym*: indicates that the symmetry of the geometry will not be used, nor that the electrons, in order to accelerate the calculations and make them more reliable; and (5) *SCF = QC*: to close the self-consistent field (SCF) when the structures do not converge. Computational calculations were performed using Wahoo, the cluster of the Centre Commun de Calcul Intensif of the Université des Antilles, Guadeloupe, France. 

### 2.6. Topological Analysis of the Electron Density by Quantum Theory of Atoms in Molecules

For a better description of the interactions present in the systems obtained by DFT reoptimization, a topological study of the electron density of the systems was performed using QTAIM [27,28]. The M06-2X functional and the 6-311++G(2df,2pd) basis functions for all atoms were used to calculate the wave function. The application of this analysis allowed us to characterize the topology of the electron density (ρ), its Laplacian $(\nabla^{2}\rho$), the kinetic (G) and potential (V) energy densities, as well as other related quantities. All these quantities were analyzed at bond critical points (BCPs), allowing us to describe and classify the molecular interactions according to Nakanishi’s criteria [32,33]. All QTAIM calculations were performed from the wave function files generated by Gaussian09 [48] with the Multiwfn 3.3.6 program [54].

## 3. Results and Discussion

### 3.1. MMH Calculations

In total, 88 systems were studied, 12 systems of SG/(H_2_O)_n=1–3_, 16 for SG/CLD/(H_2_O)_n=0–3_, 16 for SG/β-HCH/(H_2_O)_n=0–3_, 12 for SG^+^/(H_2_O)_n=1–3_, 16 for SG^+^/CLD/(H_2_O)_n=0–3_, and 16 for SG^+^/β-HCH/(H_2_O)_n=0–3_. 

Because the adsorption of CLD and β-HCH occurs in aqueous solution and they co-exist in real water, it is important to understand the role that water molecules play in the interaction between both pollutants with the nitrogen-containing surface groups of AC. In previous work, the interaction of water molecules with acidic surface groups in two different models of AC, naphthalene [23] and coronene [29,30], was studied. This allowed the authors to determine that the minimum number of water molecules necessary to saturate the surface groups of the AC is three. This criterion was taken into account to compare the present results with those obtained by Gamboa-Carballo et al. [29] and Melchor-Rodríguez et al. [30], who evaluated the adsorption process of CLD onto AC but with acidic SG of activated, using the same PM7 semiempirical Hamiltonian. 

**Figure 3:**
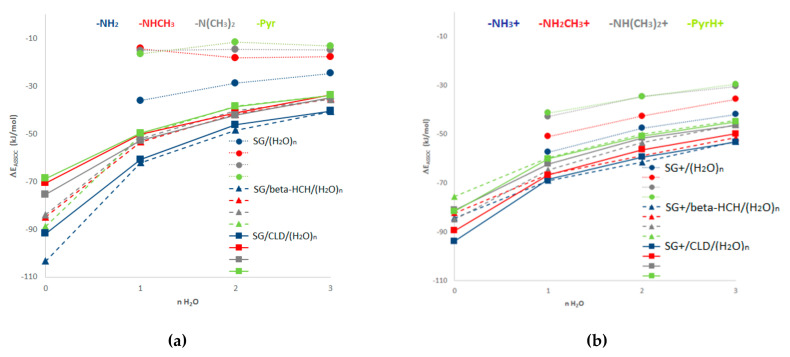
Thermodynamic association energy (ΔE_ASSOC_) for the systems SG/(H_2_O)_n=1–3_, SG/β-HCH/(H_2_O)_n=0–3_ and SG/CLD/(H_2_O)n_=0–3_ including the charged forms of SG. (**a**) Neutral and basic pH condition (β-HCH and CLD molecule and neutral SG); (**b**) acidic pH condition (β-HCH and CLD molecule and protonated SG).

Figure 3 shows the mean association values of the systems SG/(H_2_O)_n=1–3_ (dotted lines), SG/β-HCH/(H_2_O)_n=0–3_ (striped lines), and SG/CLD/(H_2_O)_n=0–3_ (solid lines) obtained by MMH procedures.

As shown in Figure 3a, the association of water molecules is greater with -NH_2_ compared to -NHCH_3_, -N(CH_3_)_2_ and -Pyr, with the lowest -NH_2_ values obtained when a single molecule of water was added. For the rest of the SGs, the saturation corresponds to an aggregate containing two or three water molecules. For the groups -NHCH_3_, -N(CH_3_)_2_ and -Pyr, the saturation behaves similarly. This behavior can also be observed in the coronene/(H_2_O)_n=1–3_ systems (Table S1, Supplementary Materials). However, no significant differences are observed in the ΔE_ASSOC_ (for the four SG in neutral and basic conditions) when the number of water molecules increases. These results indicate that the first water molecule saturates the -NH_2_ group, and this is decisive for the solvation processes. On the other hand, under acidic pH conditions, where the nitrogen-containing SGs are protonated (SGs^+^/(H_2_O)_n=1–3_), the ΔE_ASSOC_ decreases for all the systems, but the -NH3^+^/(H_2_O)_n=0–3_ and -NH_2_CH_3_^+^/(H_2_O)_n=0–3_ show the lowest values; and one molecule of water is enough to saturate the SGs. This fact can be explained, in part, based on resonance contributions to the overall hybrid of an arylamine. In neutral amines, the delocalization of the unshared electron pair of the nitrogen atom over the benzene ring (extended to coronene molecules) stabilizes the amine molecules. This delocalization of the electron pair makes it less available to a proton, and delocalization of the electron pair stabilizes the arylamines (primary > tertiary amine (methyl groups enhance the stability) > secondary amine). However, once the electron pair of the nitrogen atom accepts the proton (protonated amines), it is no longer available to participate in resonance. Therefore, the hydrogen atoms of amine groups have more lability and can interact with greater strength with the water molecules stabilizing the systems. In this sense, the primary and secondary amines (-NH_2_, -NHCH_3_), compared to the tertiary amine (-N(CH_3_)_2_), have more labile hydrogen atoms to form a hydrogen bond with the water molecules. In the specific case of pyridine, the nitrogen atom has an unshared electron pair in an sp2 orbital that is not part of the aromatic system. For that reason, under neutral and basic pH conditions, more than one water molecule is needed to saturate this SG. However, its lower ΔE_ASSOC_ values, compared to amines (at all pH ranges), can be explained by the fact that pyridine is more basic than amines (aminium ion pK_a_ of primary amine: 4.58; aminium ion pK_a_ of pyridine: 5.23) [55].

These results are in agreement with the fact that a charged SG also increases the adsorption process with water molecules [56]. Additionally, the obtained results are consistent with those previously reported qualitatively by Gamboa-Carballo et al. [29] and Melchor-Rodríguez et al. [30] when they evaluated the interactions of CLD with acidic SG (COOH and OH) using the same level of theory for MMH-PM7 calculations. The study with basic pH yields similar results to those obtained with neutral pH. 

On the other hand, comparing the mean association energy values of every system in the presence of contaminants (SG/β-HCH and SG/CLD) with the corresponding association of the AC model with water molecules alone (SG/(H_2_O)_n=1–3_), the first ones were considerably lower. On the other hand, the stability of the systems SG/pesticides was considerably higher than those SG/pesticides/(H_2_O)_n=1–3_ (although these were still energetically favored). This behavior suggests that if CLD or β-HCH molecules are associated with the SGs, the molecules of water would not be able to compete for the adsorption sites and easily displace the pesticides. However, it is important to point out that in real systems, both pesticides are much diluted and the adsorption constant would be displaced due to this fact. The numerical values of ΔE_ASSOC_ for SG/(H_2_O)_n=0–3_) and SG/pesticides/(H_2_O)_n=1–3_ systems are shown in Tables S1 and S2 (Supplementary Materials). 

Finally, considering the ΔE_ASSOC_ values that are shown in Figure 3, for the SG/pesticides/(H_2_O)_n=0–3_ and SG^+^/pesticides/(H_2_O)_n=0–3_ systems, it can be observed how pH influences the stability of the association complexes with the nitrogen-containing surface groups. For both pollutants, more stable association complexes are obtained under acidic pH when the surface group is charged. Thus, it can be concluded that neutral and basic pH levels are the less favorable condition for the adsorption of the pesticides under study. In fact, the removal of 2,4-dichlorophenol and benzoic acid from water employing nitrogen-containing SGs onto natural synthetized AC was observed to be highly dependent on the pH of the solution, which affects the surface charge of the AC [57,58]. In those works, the highest percentage of removal of both pollutants w achieved at lower pH (nitrogen-containing SGs are charged), and on the contrary, an increase in the pH solution decreased the removal efficiency.

**Figure 4:**
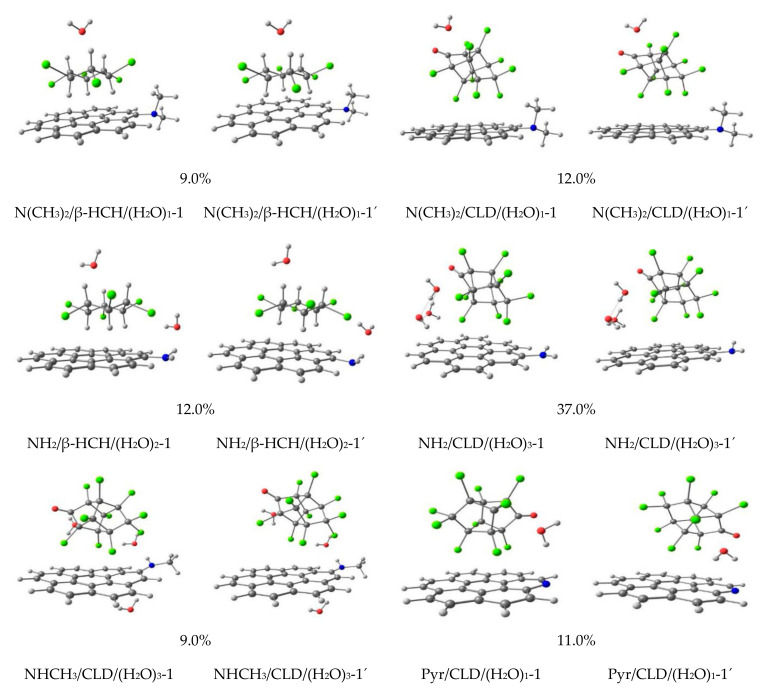
Distinctive minima structures and their population (%) under neutral and basic pH conditions. Numbers denote the most stable structures found at MMH-PM7 calculations, while quoted numbers account for the corresponding reoptimized structures at the MO6-2X/6-31+G(d,p) level of theory.

**Figure 5:**
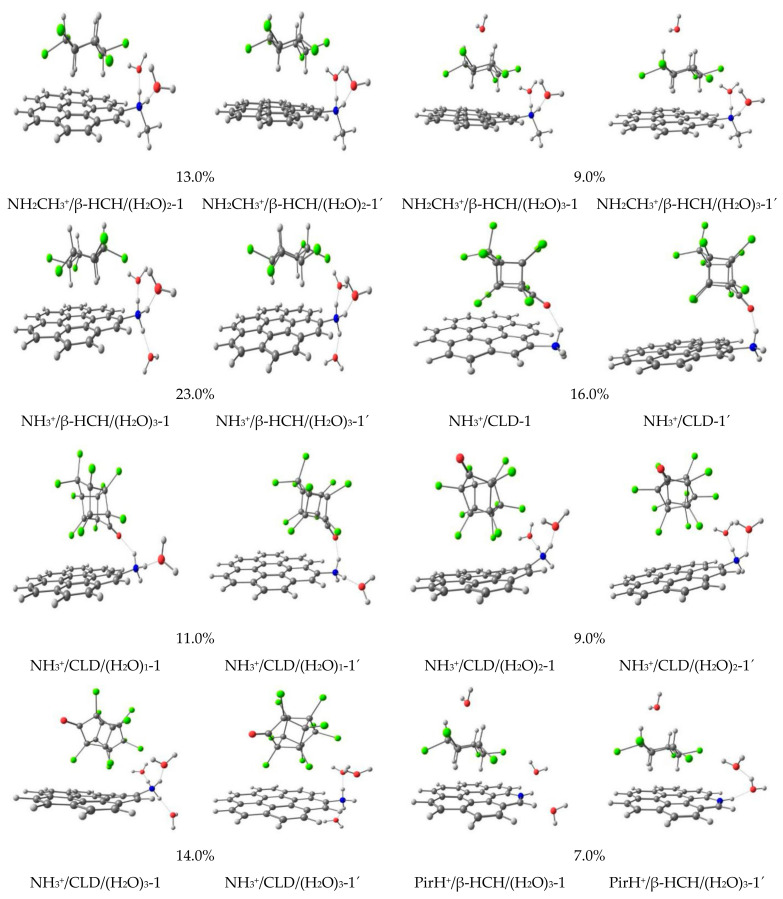
Distinctive minima structures and their population (%) underacidic pH conditions. Numbers denote the most stable structures found at MMH-PM7 calculations, while quoted numbers account for the corresponding reoptimized structures at the MO6-2X/6-31+G(d,p) level of theory.

Figure 4 and Figure 5 present some of the structures obtained by MMH-PM7 calculations under neutral, basic, and acidic pH conditions. Two distinctive interactions were obtained for the systems under study. The first interaction type consists of the chlorine atoms of CLD and β-HCH pesticides interacting with the carbon atoms of coronene molecules (Cl· · ·C). This kind of interaction is the most abundant one after the analysis of the structure of each complex. In addition, this interaction is also observed between the axial hydrogens of β-HCH and the graphitic surface of coronene (H···C). The second interaction type is an electrostatic interaction through H-bond between the water molecules with: a) ketone groups of CLD and the β-HCH: N(CH_3_)_2_/CLD/(H_2_O)_1_-1 and (N(CH_3_)_2_/β-HCH/(H_2_O)_1_-1; b) with the nitrogen-containing surface groups: NH_3_/CLD/(H_2_O)_2_-1; NH_2_CH_3_^+^/β-HCH/(H_2_O)_2_-1; NH_3_^+^/β-HCH/(H_2_O)_3_-1; and c) forming water clustering: (NH_2_/CLD/(H_2_O)_3_-1. Moreover, an electrostatic interaction was also found between the protonated primary amine surface group and CLD (NH^3+^/CLD-1 and NH^3+^/CLD/(H_2_O)_1_-1) (Figure 5). Note that only these two complexes show this type of interaction, where the pesticide interacts directly with the nitrogen-containing surface groups. These interactions are further described through QTAIM results.

To summarize, according to the mean association energy values obtained for each system under study, the affinity order between the water molecules and the SG is: -NH_2_ > -NHCH_3_ ≈ -N(CH_3_)_2_ ≈ -Pyr > coronene. For the systems with CLD, the affinity order is: NH_3_^+^/CLD/(H_2_O)_n=0–3_ > NH_2_CH_3_^+^/CLD/(H_2_O)_n=0–3_ > NH(CH_3_)_2_^+^/CLD/(H_2_O)_n=0–3_ ≈ PirH^+^/CLD/(H_2_O)_n=0–3_. Additionally, for β-HCH, the affinity order is: NH_3_^+^/β-HCH/(H_2_O)_n=0–3_ > NH_2_CH_3_^+^/β-HCH/(H_2_O)_n=0–3_ > NH(CH_3_)_2_^+^/β-HCH/(H_2_O)_n=0–3_ ≈ PirH^+^/β-HCH/(H_2_O)_n=0–3_. The electrostatic and dispersive interactions are present in most of the structures obtained by the MMH methodology. This behavior is in agreement with the fact that surface chemistry and solution pH are the most important factors controlling the adsorption process. In this case, dispersive interactions are predominant, mainly because of the attraction between the π orbitals on the carbon basal planes (π-cloud) and the pollutants. However, when the solution pH is very high or very low, electrostatic interactions between the pollutants and charged functional groups on the carbon surface could be significant [53].

### 3.2. Influence of the Surface Group’s Nature 

**Figure 6:**
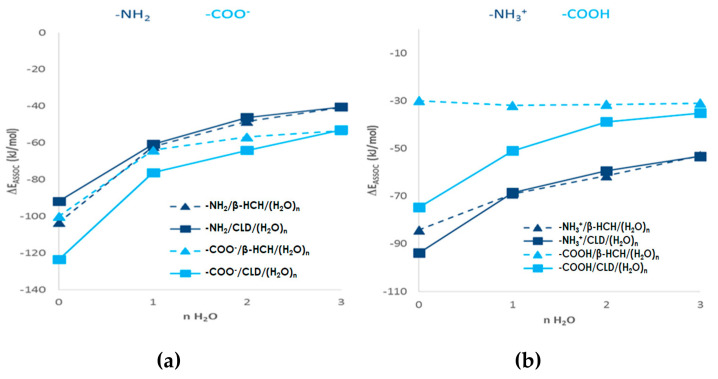
Influence of the surface group nature in AC under different pH conditions (**a**) neutral and basic pH, (**b**) acidic pH. The ΔE_ASSOC_ are obtained by MMH-PM7.

The role of the nature of the acidic (previous results by our researcher group) [29,30], and basic nitrogen-containing SGs (present results) in the adsorption of CLD (pesticide that showed the best ΔEASSOC values (compared to β-HCH) was evaluated. Figure 6a,b show the mean association energy values of the best acidic surface groups (COOH) [29,30] and basic nitrogen-containing surface groups (NH2) interacting with CLD under neutral, basic, and acidic pH conditions. In both cases, the results were obtained with the same level of theory, PM7 semiempirical Hamiltonian coupled with MMH methodology. As can be observed, the deprotonated carboxyl groups (COO-) show the best results under neutral and basic conditions compared to the arylamine group (Figure 6a). However, the best results were obtained at pH ≈ 5–9, where only COOH groups were deprotonated to a considerable extent (≈90%). Gamboa-Carballo et al. [29], by QTAIM results, confirmed the presence of covalent interactions between the negatively charge oxygen of the (COO- and O-) acidic surface groups and the carbonylic carbon of the CLD, further suggesting chemisorption between CLD and the charged SGs, especially for COO- under slightly acidic and neutral pH conditions. On the other hand, with acidic pH, the best results were obtained for the primary amine compared to COOH groups (Figure 6b). This behavior may be due to the protonated amine, which should enhance the H-bonding interaction between the ketone group and the H labile atoms of the SG in both the absence and presence of water molecules, as can be observed in Figure 5 (NH_3_^+^/CLD-1 and NH_3_^+^/CLD/(H_2_O)1-1 systems). Additionally, with acidic pH, the COOH surface group is in neutral form, and the results by Gamboa-Carballo et al. [29] suggest a non-dependence of the association energy with acidic SGs at lower pH.

**Figure 7:**
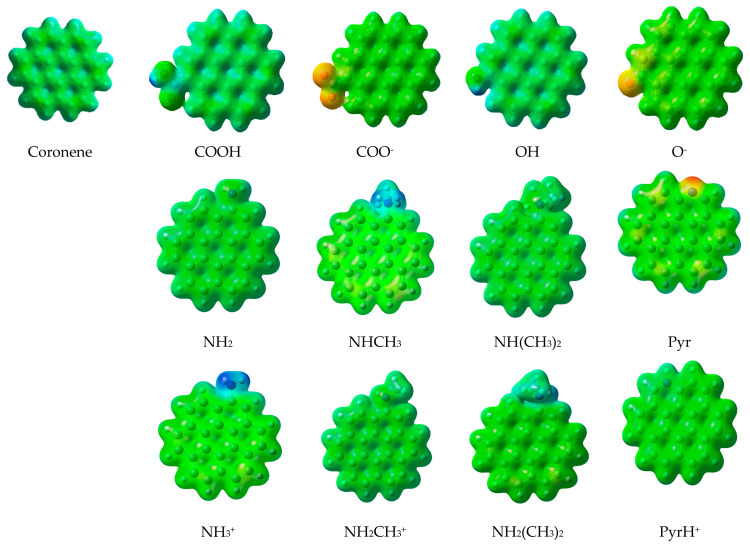
Maps of the total electrostatic potential for coronene, coronene-acidic surface groups (COOH and OH), and their deprotonated forms (COO- and O-); and for coronene-nitrogen-containing basic surface groups (primary, secondary, and tertiary amine and pyridine) and their protonated forms. (Red areas are more negative and blue areas are less negative).

In order to corroborate the above results, the total electrostatic potentials (ESP) for acidic surface groups (COOH and OH) and their deprotonated forms, as well as basic nitrogen-containing surface groups (primary, secondary and tertiary amines, and pyridine) and their protonated forms, were mapped using the program Multiwfn 3.3.6 [59]. Likewise, the total electrostatic potential of the coronene molecule was mapped to be used as reference. The results of this mapping of the total ESP are shown in Figure 7. Here, the isosurface contour value of 0.002 a.u. was chosen to account for the reactive surface of the studied molecules. The total ESP values range from −0.4 a.u. (blue) to 0.4 a.u. (red) for -COOH, -OH, -COO^−^, -O-, and coronene; and from −0.1 a.u. (blue) to 0.4 a.u. (red) for -NH_2_; -NHCH_3_,-N(CH_3_)_2_, -Pyr, and its protonated forms; light blue, green, and yellow, respectively, represent the progressive increment of the total ESP between these two limits. 

As can be seen, when acidic or basic nitrogen-containing surface groups are added to the coronene molecule, a surface activation occurs. For acidic SGs, this behavior is more remarkable for COO- and O-, demonstrating that interactions are favored with the charged hydroxyl and carboxyl surface groups. This indicates that the sorption will be more favorable at the pH intervals where the surface groups are deprotonated (pH > 5 for the carboxyl group and pH > 8 for the hydroxyl group). This result ratifies the idea of chemical sorption at slightly acidic and neutral pH conditions of CLD with COO-, as was obtained by Gamboa-Carballo et al. [29]. Additionally, this reinforces the experimental results previously obtained by Durimel et al. [22] through desorption studies showing that carboxylic groups on the AC play a major role in CLD adsorption. For amines, fewer electronegative groups were obtained, highlighting the second amine and the protonated primary amine. These results agree with what has been explained above in the section on MMH calculations.

### 3.3. DFT Reoptimization by M06-2X/6-31+G(d,p)

The reoptimization of structures previously selected from MMH results was carried out using DFT to achieve a better characterization of the molecular geometry, the strength, and the nature of the present interactions in the systems under study. A total of 14 minima distinctive structures of the SG/pesticide/(H_2_O)_n=0–3_ and SG^+^/pesticide/(H_2_O)_n=0–3_ systems were selected. The selection criterion was based on the structures with the predominant interactions and those with the highest probability according to the Boltzmann energy distribution. Six structures were selected for neutral and basic pH, and eight structures for acidic pH. Of the total structures selected, eight correspond to the CLD molecule, and six structures correspond to the β-HCH molecules. This reoptimization describes the significant structures on a more rigorous level of theory. 

Figure 4 and Figure 5 show the 14 obtained geometries of the global MMH minima after being reoptimized using DFT (figures identify by quoted numbers). As can be seen, in general, all structures keep their initial geometries. Thus, the reoptimized geometries do not differ significantly from the ones obtained from the semiempirical optimization by PM7. However, some differences for the Cl···C interactions were observed in the supermolecules, increasing the interactions between the chlorine atoms of the pesticides and the carbon atoms of the graphene surface (Pyr/CLD/(H_2_O)_1_-1′ and NH_3_^+^/CLD-1′). This could be taken as a signal of the importance of this kind of interaction in the stabilization of the studied systems. Moreover, it can be assumed that PM7 overestimates this kind of dispersive interaction. In fact, previous works show the same behavior for Cl···C interactions [29,30,60] using the same level of theory. However, the PM7 semiempirical Hamiltonian was able to qualitatively describe the association complexes in terms of interaction types. These results demonstrate that the use of a semiempirical Hamiltonian can describe the main interaction types in the potential energy surfaces.

### 3.4. QTAIM Results 

**Figure 8:**
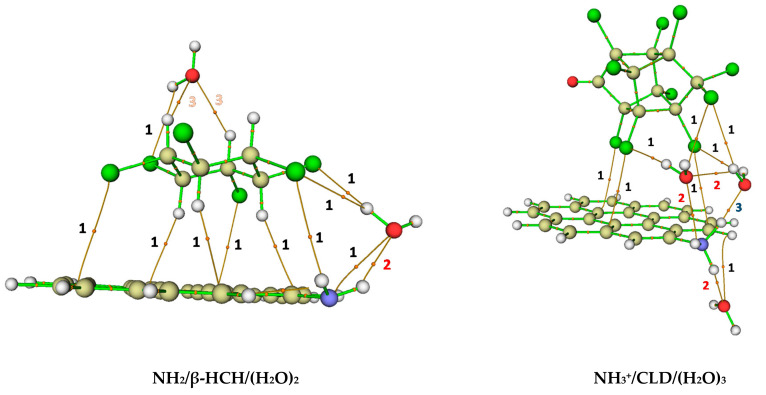
Interactions and bond paths obtained by QTAIM for distinctive minima structures of the systems NH_2_/β-HCH/(H_2_O)_2_ and NH_3_^+^/CLD/(H_2_O)_3_.

In order to carry out a more rigorous characterization of the interactions present in the complexes SG/pesticide/(H_2_O)_n=0–3_ and SG^+^/pesticide/(H_2_O)_n=0–3_, a topological analysis of the electron density was performed (Table 1 and Table 2). The application of the QTAIM analysis allowed us to characterize the topology of the electron density (ρ) and its Laplacian ($\nabla^{2}\rho$) at the bond critical points (BCPs) and, therefore, to describe the molecular interactions and to classify them according to generally accepted criteria. In this work, the Nakanishi’s criteria [32,33] were used for this purpose. From the analysis of the parameters characterizing the BCPs, it was possible to confirm the predominance of van der Waals dispersive interactions (76.0 %), as shown in Table 3. These interactions occur principally between the chlorine atoms of both pesticides with the carbon atoms of the coronene surface (Cl· · ·C). However, in the specific case of β-HCH, van der Waals dispersive interactions were also observed between the axial hydrogen atoms of this pesticide and the graphitic surface of coronene (H···C). Furthermore, stronger interactions like, hydrogen-bond (HB) and charge-transfer (CT) interactions can also be found in the complexes. These types of interactions were presented principally between the AC (including the functional group) and water molecules and between pesticides and water molecules, as shown in Figure 8 and Table 1 and Table 2. The QTAIM analysis performed for the representative Interactions for the systems NH_2_/β-HCH/(H_2_O)_2_ and NH_3_^+^/CLD/(H_2_O)_3_ are shown in Table 1 and Table 2. To select these structures, the presence of both pollutants and the best surface groups (NH_2_-neutral and protonated) were considered (Figure 8). The QTAIM analysis for the 12 complexes SG/pesticide/(H_2_O)_n=0–3_ and SG^+^/pesticide/(H_2_O)_n=0–3_ are presented in Table S3 (Supplementary Materials).

The presence of a large number of vdW interactions and several HBs are the forces that govern the stability of the systems under study. Table 3 shows the interactions present in all the complexes studied. As can be seen, interactions of a dispersive nature are predominant (76.0%) in all the systems, followed by HB (16.2%), HB-w (6.0%), and CT (1.8%). However, only two structures (NH_3_^+^/CLD-1 and NH_3_^+^/CLD/(H_2_O)_1_-1) show a direct interaction between the pollutant—in this case, CLD—with the charged SG through HB interaction. Moreover, an increase in the association of CLD and β-HCH with the SGs was observed when the water molecules were between both pollutants and the nitrogen-containing SGs (NH_3_^+^/CLD/(H_2_O)_3_; NH_2_CH_3_^+^/β-HCH/(H_2_O)_2_). This behavior principally occurs when the SGs are charged. In addition, only at acidic pH was the charge-transfer interaction type was observed between the O atom of water molecules and the H atom of surface groups, O···H interaction (NH_3_^+^/CLD/(H_2_O)_2_; NH_3_^+^/CLD/(H_2_O)_3_; Pyr^+^/β-HCH/(H_2_O)_3_). On the other hand, HB interactions (HB and HB-w) were preferentially presented in all analyzed complexes between the pesticides and SGs onto AC with water molecules. This behavior is in agreement with the fact that surface chemistry and solution pH are the most important factors controlling the adsorption process. In the present case, dispersive interactions are predominant mainly because of the attraction between the graphitic surface of coronene molecules and the pollutants (Cl···C and H···C). However, when the solution pH is very high or very low, electrostatic interactions (HB) between the pollutants and charged functional groups on the carbon surface could be significant [56]. Actually, basic nitrogen-containing surface groups of AC have been used to remove heavy metals [61] and organic compounds [57,62,63] from aqueous media. The nitrogen-containing functional groups are introduced onto the AC surface in order to increase the adsorption capacity, selectivity, and removal efficiency of these pollutants due to the strengthened π–π dispersion force, as well as the coordination of surface functional groups [62]. Regarding that, Qin et al. [58] evaluated the adsorption of benzoic acid from aqueous solution employing nitrogen-containing SG onto AC. In that work, the enhancement of adsorption capacity was due to the strengthened π–π dispersion force between benzoic acid and the AC basal plane, and it was found that the highest adsorption occured at pH 3.8 [58]. 

To summarize, van der Waals and electrostatic interactions confer stability to the complexes formed between the nitrogen-containing functionalized activated carbon, the pollutants, and water molecules. This is in agreement with MMH results and is consistent with the QTAIM analyses. Therefore, these results suggest that the adsorption process of CLD and β-HCH under different pH and solvation conditions onto nitrogen basic SGs of AC occurs through a physical mechanism. 

Finally, when comparing the ΔEASSOC values of the best basic nitrogen-containing SG with the best acidic SG (COOH), the carboxyl group (COO^−^) showed the greatest results under neutral and basic pH conditions. For that reason, it can be concluded that the acidic surface group showed the best results. As was explained previously, Gamboa-Carballo et al. [29] found a covalent interaction between CLD and COO^−^ SG (QTAIM results), further suggesting a chemisorption mechanism. However, although the presence of chemisorption was observed for the COO^−^ group with the CLD at pH > 5–9, the present results with the basic SG under study show that the synthesis of carbons functionalized with nitrogen-containing SG should also be interesting candidates for the CLD and β-HCH removal from water with acidic pH. Moreover, their synthesis, as far as experimental work is concerned, is very similar to the synthesis of acidic carbons.

## 4. Conclusions

1. The MMH methodology, with the simplified model of AC used in the present work, was successfully employed to explore the potential energy surfaces of the systems under study and evaluate their thermodynamic association energy under different pH conditions. On the other hand, regarding hydration effects, it was observed that one water molecule saturated the primary amine (neutral and protonated) SG compared to the rest, where the saturation corresponds to an aggregate containing two or three water molecules. The same behavior was observed for the systems in presence of the pesticides. 

2. The mean association energy values demonstrated that the complexes with acidic surface groups are more stable than complexes with basic SGs, principally with COO^−^, under neutral and basic pH conditions. 

3. When comparing the role of the nature of the basic SGs in the adsorption of the two pesticides, it was observed that the stability of the complexes is favored in the order: primary amine > secondary and tertiary amine > pyridine. However the adsorption of both pollutants is favored with acidic pH with the primary amine.

4. The reoptimization of distinctive minima structures by DFT showed, in most of the cases, that the structures conserved their geometry and the interaction types. 

5. The performed calculations by QTAIM showed that the association of CLD and β-HCH with basic SGs occurs preferentially due to the interaction of the chlorine of the CLD and the β-HCH with the graphitic surface of coronene. Additionally, hydrogen-bond-type electrostatic interactions were observed when water molecules were added to the systems. This behavior suggests that the adsorption process of both pollutants with the basic SGs of the AC under acidic, neutral, and basic pH conditions occurs through a physisorption mechanism.

6. The acidic surface group COO^−^ has a greater influence on the adsorption of the pesticides compared to the basic nitrogen-containing surface groups. However, the results for the basic SGs demonstrate that functionalized AC with nitrogen-containing SGs can be used as possible candidates for CLD and β-HCH removal from water with acidic pH.

## Figures and Tables

**Table 1:** Interatomic distances (d), electron density (*ρ_BCP_*), Laplacian of electron density (∇^2^*ρ_BCP_*), total energy density (*H_BCP_*), potential-kinetic energy density ratio (*V_BCP_*/*G_BCP_*), and ellipticity of the electron density (ε) at the bond critical points (BCPs) for the system NH_2_/β-HCH/(H_2_O)_2_.

Interaction Number	Atoms ^1^	d (Ȧ)	$\rho_{BCP}$ $\left(ea_{0}^{-3}\right)$	$\nabla^{2}\rho_{BCP}$ $\left(ea_{0}^{-5}\right)$	$H_{BCP}$ $\left(u.a\right)$	$\frac{V_{BCP}}{G_{BCP}}$	ε	Interaction Type ^2,3^
1	O42…H19 (H_2_O…AC)	2.77	0.0069	0.023	0.0007	−0.85	0.17	vdW
2	O42…H37 (H_2_O…AC)	2.06	0.0189	0.078	0.0026	−0.85	0.02	HB
1	Cl62…C14 (P…AC)	3.47	0.0067	0.022	0.0011	−0.75	3.01	vdW
1	H44...Cl60 (H_2_O…P)	2.61	0.0100	0.034	0.0015	−0.78	0.13	vdW
1	H44...Cl59 (H_2_O…P)	2.72	0.0080	0.029	0.0014	−0.75	0.18	vdW
1	Cl59…N36 (P…AC)	3.47	0.0072	0.023	0.0009	−0.80	0.73	vdW
1	H51…C22 (P…AC)	2.70	0.0082	0.027	0.0011	-0.80	8.21	vdW
1	Cl58…C27 (P…AC)	3.51	0.0068	0.021	0.0010	−0.76	2.63	vdW
1	H55…C9 (P…AC)	2.69	0.0079	0.026	0.0012	−0.78	4.95	vdW
1	H53…C6 (P…AC)	2.66	0.0091	0.030	0.0012	−0.81	3.79	vdW
3	O39…H54 (H_2_O…P)	2.28	0.0134	0.050	0.0017	−0.85	0.10	HB-w
3	O39…H56 (H_2_O…P)	2.29	0.0133	0.049	0.0016	−0.85	0.09	HB-w
1	H40…Cl61 (H_2_O…P)	2.66	0.0099	0.036	0.0015	−0.79	0.12	vdW

^1^Atoms interacting (above) and molecules interacting (below). AC: activated carbon; P: pesticide; H_2_O: water molecule. ^2^ The interactions were classified according to Nakanishi’s criteria [49,50]. ^3^ vdW: van der Waals dispersive interaction; HB: hydrogen bond; HB-w: hydrogen bond weak; CT: charge transfer.

**Table 2:** Interatomic distances (d), electron density (*ρ_BCP_*), Laplacian of electron density (∇^2^*ρ_BCP_*), total energy density (*H_BCP_*), potential-kinetic energy density ratio (*V_BCP_*/*G_BCP_*), and ellipticity of the electron density (ε) at the bond critical points (BCPs) for the system NH_3_^+^/CLD/(H_2_O)_3_.

Interaction Number	Atoms ^1^	d (Ȧ)	$\rho_{BCP}$ $\;(ea_{0}^{-3})$	$\nabla^{2}\rho_{BCP}$ $\left(ea_{0}^{-5}\right)$	$H_{BCP}$ $\left(u.a\right)$	$\frac{V_{BCP}}{G_{BCP}}$	ε	Interaction Type ^2,3^
1	O64…H25 (H_2_O…AC)	2.81	0.0065	0.023	0.0009	−0.83	0.44	vdW
2	O64…H38 (H_2_O…AC)	1.81	0.0333	0.111	−0.0011	−1.04	0.06	HB
1	Cl46…C12 (P…AC)	3.20	0.0090	0.032	0.0015	−0.76	1.62	vdW
1	Cl60…C1 (P…AC)	3.42	0.0069	0.022	0.0011	−0.76	1.73	vdW
1	Cl60…H69 (P…H_2_O)	2.76	0.0076	0.026	0.0012	−0.76	0.16	vdW
3	O67…H39 (H_2_O…AC)	1.63	0.0574	0.100	−0.0176	−1.41	0.03	CT
1	H68…Cl59 (H_2_O…P)	2.71	0.0087	0.030	0.0013	−0.79	0.25	vdW
2	H68…O61 (H_2_O…H_2_O)	2.08	0.0191	0.076	0.0021	−0.87	0.20	HB
1	O61…Cl59 (H_2_O…P)	3.36	0.0070	0.024	0.0009	−0.81	0.51	vdW
2	O61…H37 (H_2_O…AC)	2.36	0.0128	0.050	0.0018	−0.83	0.51	HB
1	H62…Cl47 (H_2_O…P)	2.53	0.0110	0.038	0.0016	−0.80	0.04	vdW
1	Cl47…C3 (P…AC)	3.29	0.0081	0.027	0.0013	−0.76	1.08	vdW

^1^ Atoms interacting (above) and molecules interacting (below). AC: activated carbon; P: pesticide; H_2_O: water molecule. ^2^ The interactions were classified according to Nakanishi’s criteria [49,50].^3^ vdW: van der Waals dispersive interaction; HB: hydrogen bond; CT: charge transfer.

**Table 3:** Interaction types (%) present in the 14 distinctive minima structures by QTAIM results and using the Nakanishi criteria.

No.	Complex	Interaction Types			
		vdW	HB	HBw	CT
1	NH_3_^+^/β-HCH/(H_2_O)_3_	10 (71.4%)	3 (21.4%)	1 (7.1%)	-
2	NH_2_CH_3_^+^/β-HCH/(H_2_O)_2_	11 (73.3%)	2 (13.3%)	2 (13.3%)	-
3	NH_2_CH_3_^+^/β-HCH/(H_2_O)_3_	13 (64.4%)	4 (21.0%)	2 (10.5%)	-
4	PyrH^+^/β-HCH/(H_2_O)_3_	9 (64.3%)	3 (21.4%)	1 (7.1%)	1 (7.1%)
5	NH_3_^+^/CLD	5 (83.3%)	1 (16.6%)	-	-
6	NH_3_^+^/CLD/(H_2_O)_1_	6 (75.0%)	2 (25.0%)	-	-
7	NH_3_^+^/CLD/(H_2_O)_2_	8 (72.7%)	1 (9.1%)	1 (9.1%)	1 (9.1%)
8	NH_3_^+^/CLD/(H_2_O)_3_	8 (66.6%)	3 (25.0%)	-	1 (8.3%)
9	NH_2_/β-HCH/(H_2_O)_2_	10 (76.9%)	1 (7.7%)	2 (15.4%)	-
10	N(CH_3_)_2_/β-HCH/(H_2_O)_1_	9 (81.8%)	2 (18.2%)	-	-
11	NH_2_/CLD/(H_2_O)_3_	10 (71.4%)	4 (28.6%)	-	-
12	NHCH_3_/CLD/(H_2_O)_3_	12 (85.7%)	1 (7.1%)	1 (7.1%)	-
13	N(CH_3_)_2_/CLD/(H_2_O)_1_	9 (100.0%)	-	-	-
14	Pyr/CLD/(H_2_O)_1_	7 (100.0%)	-	-	-
	Total	127 (76.0%)	27 (16.2%)	10 (6.0%)	3 (1.8%)

## Data Availability

The data presented in this study are available in supplementary material.

## Supplementary Materials

The following are available online. Table S1. Mean association energy of the systems SG/(H_2_O)n=1–3. SG/CLD/(H_2_O)n=0–3 and SG/β-HCH/(H_2_O)n=0–3 at neutral and basic pH conditions. The values are reported in kJ/mol, Tabla S2. Mean association energy of the systems SG+/(H_2_O)n=1–3, SG+/CLD/(H_2_O)n=0–3 and SG+/β-HCH/(H_2_O)n=0–3 (acidic pH). The values are reported in kJ/mol, Table S3. Interatomic distances (d). electron density (*ρ_BCP_*). Laplacian of electron density (∇2*ρ_BCP_*). total energy density (*H_BCP_*). potential-kinetic energy density ratio (*V_BCP_*/*G_BCP_*) and ellipticity of the electron density (ε) at the bond critical points (BCPs) for the systems SG/CLD/(H_2_O)n=0–3 and SG/β-HCH/(H_2_O)_n=0–3_ using the Nakanshi criteria.
