# Supplementary material for: Role of Basic Surface Groups of Activated Carbon in Chlordecone and β-Hexachlorocyclohexane Adsorption: A Molecular Modelling Study
Source: Molecules. 2021 Nov 18;26(22):6969. doi: 10.3390/molecules26226969 (PMC8619540; doi:10.3390/molecules26226969)
Supplement: Supplementary file 1 [file molecules-26-06969-s001.zip › molecules-1455132-supplementary.pdf]

Supplementary Materials: Role of basic surface groups of activated carbon in chlordecone and β-hexachlorocyclohexane adsorption. molecular modelling study.

Table S1. Mean association energy of the systems SG/$(H_2O)_{n=1-3}$, SG/CLD/$(H_2O)_{n=0-3}$ and SG/β-HCH/$(H_2O)_{n=0-3}$ at neutral and basic pH conditions. The values are reported in kJ/mol.

n/GS	NH_2	$NHCH_3$	$N(CH_3)_2$	Pyr
**No pesticide**				
**1**	-36.1	-14.4	-15.2	-16.5
**2**	-28.8	-18.3	-14.7	-11.6
**3**	-24.7	-17.7	-14.9	-13.3
**CLD**				
**0**	-91.8	-70.6	-75.5	-68.7
**1**	-60.8	-50.1	-52.8	-49.7
**2**	-46.2	-41.2	-42.2	-38.5
**3**	-40.5	-33.7	-34.8	-33.9
**β-HCH**				
**0**	-103.4	-84.9	-83.9	-88.8
**1**	-62.1	-53.5	-52.0	-50.7
**2**	-48.5	-41.8	-40.4	-38.4
**3**	-40.8	-35.3	-35.6	-33.9

Tabla S2. Mean association energy of the systems $SG^+/(H_2O)_{n=1-3}$, SG^+/CLD/$(H_2O)_{n=0-3}$ and SG^+/β-HCH/$(H_2O)_{n=0-3}$ (acidic pH). The values are reported in kJ/mol.

n/GS	NH_3^+	$NH_2CH_3^+$	$NH(CH_3)_2^+$	$PyrH^+$
**No pesticide**				
**1**	-57.3	-51.0	-42.8	-41.3
**2**	-47.5	-42.7	-34.6	-34.7
**3**	-42.0	-35.7	-30.4	-29.6
**CLD**				
**0**	-93.8	-89.5	-81.1	-81.6
**1**	-68.5	-66.7	-62.2	-60.1
**2**	-59.4	-56.4	-51.6	-50.8
**3**	-53.3	-50.4	-46.4	-44.9
**β-HCH**				
**0**	-84.2	-82.3	-84.9	-75.5
**1**	-69.1	-66.5	-64.6	-59.6
**2**	-61.5	-58.9	-53.4	-50.1
**3**	-53.0	-51.4	-46.2	-44.5

Table S3. Interatomic distances (d), electron density (ρ_{BCP}), Laplacian of electron density ($\nabla^2\rho_{BCP}$), total energy density (H_{BCP}), potential-kinetic energy density ratio (V_{BCP}/G_{BCP}), and ellipticity of the electron density (ε) at the bond critical points (BCPs) for the systems SG/CLD/$(H_2O)_{n=0-3}$ and SG/β-HCH/$(H_2O)_{n=0-3}$ using the Nakanishi's criteria.

$NH_2CH_3^+$/β-HCH/$(H_2O)_2$

Interactions	Atoms[1]	d (A)	ρ_{BCP} (ea_0^{-3})	$\nabla^2\rho_{BCP}$ (ea_0^{-5})	H_{BCP} ($u.a$)	$\frac{V_{BCP}}{G_{BCP}}$	ε	Interaction type[2,3]
**1**	C16…Cl56 (P…AC)	3.54	0.0061	0.019	0.0010	-0.75	5.81	vdW
**2**	C10…H53 (P…AC)	2.76	0.0072	0.024	0.0010	-0.79	13.08	vdW
**3**	C5…H49 (P…AC)	2.71	0.0081	0.026	0.0011	-0.80	2.93	vdW
**4**	C1…Cl60 (P…AC)	3.51	0.0070	0.021	0.0010	-0.78	2.29	vdW
**5**	H65…Cl60 (H_2O…P)	2.56	0.0011	0.004	0.0000	-0.80	0.03	vdW
**6**	H25…O64 (H_2O…AC)	2.44	0.0114	0.042	0.0014	-0.84	0.02	HB-w
**7**	H38…O64 (H_2O…AC)	1.77	0.0386	0.105	-0.0047	-1.15	0.05	HB
**8**	O64…Cl58 (H_2O…P)	3.34	0.0083	0.027	0.0009	-0.85	0.56	vdW
**9**	C13…H51 (P…AC)	2.60	0.0103	0.034	0.0013	-0.82	1.81	vdW
**10**	H37…O61 (H_2O…AC)	1.79	0.0354	0.109	-0.0025	-1.08	0.01	HB
**11**	H26…O61 (H_2O…AC)	2.49	0.0101	0.035	0.0011	-0.85	0.24	HB-w
**12**	H62…Cl58 (H_2O…P)	2.73	0.0084	0.029	0.0013	-0.78	0.43	vdW
**13**	H62…Cl57 (H_2O…P)	2.62	0.0097	0.034	0.0016	-0.77	0.13	vdW
**14**	C2…Cl57 (P…AC)	3.62	0.0055	0.016	0.0008	-0.76	6.05	vdW
**15**	C4…Cl57 (P…AC)	3.60	0.0055	0.016	0.0008	-0.75	4.71	vdW
**$NH_2CH_3^+$/β-HCH/$(H_2O)_3$**								
**1**	Cl58...C16 (P…AC)	3.54	0.0061	0.019	0.0010	-0.75	7.29	vdW
**2**	Cl58...C17 (P…AC)	3.54	0.0060	0.019	0.0010	-0.75	9.37	vdW
**3**	O67…H52 (H_2O…P)	2.29	0.0137	0.049	0.0016	-0.85	0.12	HB
**4**	O67…H54 (H_2O…P)	2.23	0.0147	0.056	0.0019	-0.84	0.14	HB

**5**	O67…Cl60 (H_2O…P)	3.36	0.0068	0.025	0.0010	-0.80	0.48	vdW
**6**	H57…C5 (P…AC)	2.71	0.0083	0.027	0.0011	-0.80	3.00	vdW
**7**	H53…C10 (P…AC)	2.80	0.0068	0.022	0.0010	-0.78	8.97	vdW
**8**	Cl61…C1 (P…AC)	3.51	0.0070	0.021	0.0010	-0.78	2.23	vdW
**9**	H65…Cl61 (H_2O…P)	2.53	0.0111	0.037	0.0015	-0.81	0.03	vdW
**10**	O64…H25 (H_2O…AC)	2.43	0.0115	0.043	0.0015	-0.84	0.02	HB-w
**11**	O64…H38 (H_2O…AC)	1.77	0.0390	0.104	-0.0050	-1.16	0.05	HB
**12**	O64…Cl63 (H_2O…P)	3.32	0.0085	0.028	0.0009	-0.85	0.55	vdW
**13**	O43…H37 (H_2O…AC)	1.79	0.0354	0.109	-0.0025	-1.08	0.07	HB
**14**	O43…H26 (H_2O…AC)	2.48	0.0103	0.036	0.0011	-0.85	0.23	HB-w
**15**	H45…Cl63 (H_2O…P)	2.72	0.0087	0.030	0.0013	-0.79	0.44	vdW
**16**	H45…Cl62 (H_2O…P)	2.60	0.0100	0.034	0.0016	-0.78	0.13	vdW
**17**	Cl62…C2 (P…AC)	3.63	0.0054	0.016	0.0008	-0.76	4.71	vdW
**18**	Cl62…C4 (P…AC)	3.61	0.0054	0.016	0.0008	-0.75	4.46	vdW
**19**	H55…C13 (P…AC)	2.60	0.0104	0.034	0.0013	-0.82	1.81	vdW
**NH_3^+/β-HCH/$(H_2O)_3$**								
**1**	O61…H38 (H_2O…AC)	1.82	0.0326	0.111	-0.0007	-1.02	0.06	HB
**2**	O40…H25 (H_2O…AC)	2.38	0.0119	0.041	0.0012	-0.87	0.14	HB-w
**3**	O40…H39 (H_2O…AC)	1.89	0.0273	0.096	0.0004	-0.98	0.09	HB
**4**	O40…Cl57 (H_2O…P)	3.08	0.0107	0.041	0.0015	-0.83	0.79	vdW
**5**	Cl57…H39 (P…AC)	2.79	0.0085	0.030	0.0013	-0.80	0.57	vdW
**6**	O64…Cl57 (H_2O…P)	3.39	0.0072	0.025	0.0009	-0.82	0.60	vdW
**7**	O64…H26 (H_2O…AC)	2.52	0.0098	0.034	0.0011	-0.85	0.24	vdW
**8**	H65…Cl55 (H_2O…P)	2.56	0.0106	0.036	0.0015	-0.80	0.08	vdW
**9**	H49…C3 (P…AC)	2.62	0.0093	0.031	0.0013	-0.80	3.89	vdW
**10**	Cl56…C6 (P…AC)	3.46	0.0068	0.022	0.0011	-0.75	2.08	vdW
**11**	H53…C11 (P…AC)	2.72	0.0075	0.025	0.0011	-0.78	4.15	vdW
**12**	Cl60…C24 (P…AC)	3.50	0.0066	0.021	0.0010	-0.75	2.47	vdW

**13**	O64…H37 (H_2O…AC)	1.81	0.0335	0.109	-0.0013	-1.05	0.06	HB
**14**	H51…C14 (P…AC)	2.67	0.0085	0.028	0.0012	-0.80	5.96	vdW
				**NH_3^+/CLD**				
**1**	Cl47…C8 (P…AC)	3.33	0.0079	0.026	0.0013	-0.76	2.08	vdW
**2**	Cl48…C21 (P…AC)	3.44	0.0069	0.022	0.0011	-0.74	3.43	vdW
**3**	Cl46…C13 (P…AC)	3.71	0.0051	0.014	0.0006	-0.78	1.07	vdW
**4**	O45…H39 (P…AC)	1.79	0.0356	0.107	-0.0028	-1.10	0.02	HB
**5**	O45…H25 (P…AC)	2.55	0.0096	0.034	0.0012	-0.84	0.30	vdW
**6**	Cl50…C22 (P…AC)	3.93	0.0032	0.008	0.0004	-0.74	0.68	vdW
				**NH_3^+/CLD/$(H_2O)_1$**				
**1**	Cl47…C8 (P…AC)	3.30	0.0083	0.028	0.0013	-0.76	1.57	vdW
**2**	Cl46…C13 (P…AC)	3.67	0.0054	0.015	0.0007	-0.77	0.85	vdW
**3**	O61…H26 (H_2O…AC)	2.60	0.0079	0.027	0.0009	-0.85	0.57	vdW
**4**	O61…H37 (H_2O…AC)	1.79	0.0354	0.107	-0.0028	-1.10	0.07	HB
**5**	O45…H39 (P…AC)	1.84	0.0309	0.103	-0.0006	-1.02	0.02	HB
**6**	O45…H25 (P…AC)	2.57	0.0093	0.033	0.0011	-0.84	0.32	vdW
**7**	Cl50…C22 (P…AC)	3.96	0.0030	0.008	0.0004	-0.74	0.66	vdW
**8**	Cl48…C21 (P…AC)	3.43	0.0070	0.022	0.0011	-0.74	3.18	vdW
				**NH_3^+/CLD/$(H_2O)_2$**				
**1**	O61…H25 (H_2O…AC)	2.50	0.0103	0.038	0.0013	-0.84	0.06	HB-w
**2**	O61…H39 (H_2O…AC)	1.69	0.0478	0.104	-0.0107	-1.29	0.05	CT
**3**	O61…Cl60 (H_2O…P)	3.43	0.0066	0.023	0.0009	-0.81	0.42	vdW
**4**	H63…Cl58 (H_2O…P)	2.47	0.0127	0.042	0.0015	-0.84	0.05	vdW
**5**	Cl58...C1 (P…AC)	3.37	0.0079	0.026	0.0012	-0.76	2.20	vdW
**6**	H65…Cl60 (H_2O…P)	2.92	0.0059	0.020	0.0010	-0.77	0.36	vdW
**7**	H65…Cl57 (H_2O…P)	2.65	0.0087	0.031	0.0016	-0.75	0.03	vdW
**8**	O64…H26 (H_2O…AC)	2.80	0.0073	0.027	0.0010	-0.83	1.89	vdW
**9**	O64…H37 (H_2O…AC)	1.89	0.0278	0.106	0.0060	-0.94	0.07	HB
**10**	Cl57...C3 (P…AC)	3.21	0.0091	0.032	0.0015	-0.77	1.44	vdW

**11**	Cl55...C12 (P…AC)	3.26	0.0081	0.028	0.0014	-0.76	1.26	vdW
				**NH_3^+/CLD/$(H_2O)_3$**				
**1**	O64…H25 (H_2O…AC)	2.81	0.0065	0.023	0.0009	-0.83	0.44	vdW
**2**	O64…H38 (H_2O…AC)	1.81	0.0333	0.111	-0.0011	-1.04	0.06	HB
**3**	Cl46…C12 (P…AC)	3.20	0.0090	0.032	0.0015	-0.76	1.62	vdW
**4**	Cl60…C1 (P…AC)	3.42	0.0069	0.022	0.0011	-0.76	1.73	vdW
**5**	Cl60…H69 (P…H_2O)	2.76	0.0076	0.026	0.0012	-0.76	0.16	vdW
**6**	O67…H39 (H_2O…AC)	1.63	0.0574	0.100	-0.0176	-1.41	0.03	CT
**7**	H68…Cl59 (H_2O…P)	2.71	0.0087	0.030	0.0013	-0.79	0.25	vdW
**8**	H68…O61 (H_2O…H_2O)	2.08	0.0191	0.076	0.0021	-0.87	0.20	HB
**9**	O61…Cl59 (H_2O…P)	3.36	0.0070	0.024	0.0009	-0.81	0.51	vdW
**10**	O61…H37 (H_2O…AC)	2.36	0.0128	0.050	0.0018	-0.83	0.51	HB
**11**	H62…Cl47 (H_2O…P)	2.53	0.0110	0.038	0.0016	-0.80	0.04	vdW
**12**	Cl47…C3 (P…AC)	3.29	0.0081	0.027	0.0013	-0.76	1.08	vdW
				**$PyrH^+$/β-HCH/$(H_2O)_3$**				
**1**	O61…H36 (H_2O…AC)	1.78	0.0364	0.123	-0.0015	-1.05	0.03	CT
**2**	H62…O58 (H_2O…H_2O)	1.89	0.0265	0.099	0.0010	-0.96	0.09	HB
**3**	O58…C2 (H_2O…AC)	2.99	0.0090	0.033	0.0012	-0.83	0.54	vdW
**4**	H60…Cl54 (H_2O…P)	2.54	0.0111	0.038	0.0015	-0.81	0.03	HB-w
**5**	H60…Cl53 (H_2O…P)	2.77	0.0084	0.028	0.0011	-0.81	0.31	vdW
**6**	Cl53…C4 (P…AC)	3.47	0.0069	0.022	0.0011	-0.76	3.72	vdW
**7**	H46…C9 (P…AC)	2.66	0.0083	0.029	0.0012	-0.79	3.92	vdW
**8**	H48…C15 (P…AC)	2.74	0.0077	0.025	0.0011	-0.80	2.79	vdW
**9**	Cl49…C17 (P…AC)	3.72	0.0051	0.014	0.0007	-0.76	8.62	vdW
**10**	H44…C20 (P…AC)	2.79	0.0071	0.022	0.0010	-0.79	2.21	vdW
**11**	Cl52…C24 (P…AC)	3.49	0.0067	0.021	0.0010	-0.76	4.67	vdW
**12**	O55…H43 (H_2O…P)	2.27	0.0141	0.051	0.0017	-0.85	0.13	HB
**13**	O55…H45 (H_2O…P)	2.26	0.0142	0.053	0.0018	-0.84	0.14	HB
**14**	O55…Cl51 (H_2O…P)	3.37	0.0068	0.025	0.0010	-0.80	0.46	vdW

**$N(CH_3)_2$/β-HCH/$(H_2O)_1$**								
**1**	Cl63…H43 (P…AC)	2.90	0.0065	0.021	0.0011	-0.73	0.07	vdW
**2**	Cl65…H43 (P…AC)	3.33	0.0045	0.015	0.0008	-0.72	3.26	vdW
**3**	Cl65…C1 (P…AC)	3.50	0.0070	0.021	0.0010	-0.77	2.25	vdW
**4**	H56…C22 (P…AC)	2.72	0.0082	0.027	0.0011	-0.80	3.07	vdW
**5**	H58…C4 (P…AC)	2.73	0.0076	0.025	0.0011	-0.79	10.53	vdW
**6**	Cl62…C27 (P…AC)	3.63	0.0053	0.015	0.0008	-0.75	3.18	vdW
**7**	H54…C28 (P…AC)	2.68	0.0085	0.028	0.0011	-0.80	2.27	vdW
**8**	Cl61…C34 (P…AC)	3.57	0.0066	0.021	0.0010	-0.76	3.46	vdW
**9**	O45…H55 (H_2O…P)	2.28	0.0134	0.049	0.0016	-0.85	0.10	HB
**10**	O45…H59 (H_2O…P)	2.29	0.0133	0.049	0.0016	-0.85	0.10	HB
**11**	H46…Cl60 (H_2O…P)	2.68	0.0096	0.035	0.0015	-0.79	0.14	vdW
**NH_2/β-HCH/$(H_2O)_2$**								
**1**	O42…H19 (H_2O…AC)	2.77	0.0069	0.023	0.0007	-0.85	0.17	vdW
**2**	O42…H37 (H_2O…AC)	2.06	0.0189	0.078	0.0026	-0.85	0.02	HB
**3**	Cl62…C14 (P…AC)	3.47	0.0067	0.022	0.0011	-0.75	3.01	vdW
**4**	H44...Cl60 (H_2O…P)	2.61	0.0100	0.034	0.0015	-0.78	0.13	vdW
**5**	H44...Cl59 (H_2O…P)	2.72	0.0080	0.029	0.0014	-0.75	0.18	vdW
**6**	Cl59…N36 (P…AC)	3.47	0.0072	0.023	0.0009	-0.80	0.73	vdW
**7**	H51…C22 (P…AC)	2.70	0.0082	0.027	0.0011	-0.80	8.21	vdW
**8**	Cl58…C27 (P…AC)	3.51	0.0068	0.021	0.0010	-0.76	2.63	vdW
**9**	H55…C9 (P…AC)	2.69	0.0079	0.026	0.0012	-0.78	4.95	vdW
**10**	H53…C6 (P…AC)	2.66	0.0091	0.030	0.0012	-0.81	3.79	vdW
**11**	O39…H54 (H_2O…P)	2.28	0.0134	0.050	0.0017	-0.85	0.10	HB-w
**12**	O39…H56 (H_2O…P)	2.29	0.0133	0.049	0.0016	-0.85	0.09	HB-w
**13**	H40…Cl61 (H_2O…P)	2.66	0.0099	0.036	0.0015	-0.79	0.12	vdW
**NH_2/CLD/$(H_2O)_3$**								
**1**	H62…Cl49 (H_2O…P)	3.24	0.0032	0.011	0.0006	-0.71	0.04	vdW
**2**	H62…O66 (H_2O…H_2O)	2.05	0.0200	0.077	0.0018	-0.90	0.11	HB

**3**	H61…C32 (H_2O…AC)	2.51	0.0095	0.032	0.0014	-0.79	0.58	vdW
**4**	O60…H64 (H_2O…H_2O)	1.92	0.0263	0.093	0.0007	-0.97	0.08	HB
**5**	O60…Cl54 (H_2O…P)	3.35	0.0076	0.026	0.0010	-0.83	0.26	vdW
**6**	O66…Cl54 (H_2O…P)	3.07	0.0105	0.041	0.0015	-0.83	0.31	vdW
**7**	O66…Cl56 (H_2O…P)	3.18	0.0099	0.035	0.0011	-0.85	0.71	vdW
**8**	O63…Cl54 (H_2O…P)	3.40	0.0069	0.024	0.0009	-0.82	0.23	vdW
**9**	O66…C43 (H_2O…P)	2.51	0.0198	0.079	0.0020	-0.89	0.27	HB
**10**	H65…C13 (H_2O…AC)	2.64	0.0083	0.025	0.0009	-0.84	1.15	vdW
**11**	Cl54…C9 (P…AC)	3.18	0.0093	0.033	0.0016	-0.77	1.72	vdW
**12**	Cl57…C1 (P…AC)	3.26	0.0094	0.030	0.0014	-0.78	1.50	vdW
**13**	Cl55…C24 (P…AC)	3.39	0.0069	0.023	0.0012	-0.75	1.92	vdW
**14**	H68…O63 (H_2O…H_2O)	1.78	0.0375	0.106	-0.0043	-1.14	0.03	HB
**$N(CH_3)_2$/CLD/$(H_2O)_1$**								
**1**	Cl67…H42 (P…AC)	3.25	0.0041	0.014	0.0007	-0.71	0.88	vdW
**2**	Cl66…H42 (P…AC)	3.24	0.0045	0.014	0.0007	-0.75	0.46	vdW
**3**	Cl68…H43 (P…AC)	3.23	0.0036	0.011	0.0006	-0.71	0.49	vdW
**4**	Cl68…C23 (P…AC)	3.42	0.0069	0.023	0.0011	-0.75	3.88	vdW
**5**	Cl66…C6 (P…AC)	3.35	0.0078	0.025	0.0012	-0.77	1.22	vdW
**6**	Cl65…C9 (P…AC)	3.19	0.0092	0.033	0.0016	-0.76	3.16	vdW
**7**	O45…Cl55 (H_2O…P)	3.10	0.0100	0.038	0.0014	-0.83	0.38	vdW
**8**	O45…Cl56 (H_2O…P)	3.14	0.0102	0.037	0.0012	-0.85	0.63	vdW
**9**	O45…C52 (H_2O…P)	2.52	0.0188	0.078	0.0022	-0.87	0.32	vdW
**$NHCH_3$/CLD/$(H_2O)_3$**								
**1**	Cl69…H40 (P…AC)	3.12	0.0043	0.016	0.0008	-0.74	2.05	vdW
**2**	Cl69…C1 (P…AC)	3.38	0.0066	0.024	0.0012	-0.76	3.42	vdW
**3**	Cl69…O42 (P…H_2O)	3.20	0.0090	0.032	0.0012	-0.83	0.27	vdW
**4**	Cl66…O42 (P…H_2O)	3.43	0.0072	0.025	0.0010	-0.81	1.10	vdW
**5**	Cl66…O45 (P…H_2O)	3.13	0.0094	0.036	0.0013	-0.83	0.42	vdW
**6**	O42…H25 (H_2O…AC)	2.51	0.0106	0.041	0.0015	-0.83	1.09	HB-w

**7**	O42…H37 (H_2O…AC)	2.07	0.0193	0.071	0.0016	-0.90	0.09	HB
**8**	H43…Cl68 (H_2O…P)	2.91	0.0052	0.018	0.0010	-0.70	0.15	vdW
**9**	O45…Cl68 (H_2O…P)	3.10	0.0111	0.041	0.0013	-0.86	0.87	vdW
**10**	O45…C55 (H_2O…P)	2.54	0.0187	0.075	0.0020	-0.88	0.38	vdW
**11**	Cl68…C24 (P…AC)	3.23	0.0088	0.030	0.0015	-0.76	2.85	vdW
**12**	Cl71…C4 (P…AC)	3.20	0.0096	0.033	0.0015	-0.78	0.26	vdW
**13**	H49…C4 (H_2O…AC)	2.76	0.0072	0.023	0.0008	-0.84	1.10	vdW
**14**	H50…C6 (H_2O…AC)	2.63	0.0086	0.027	0.0011	-0.80	1.10	vdW
**Pyr/CLD/(H_2O)$_1$**								
**1**	H59…N1 (H_2O…AC)	2.52	0.0088	0.031	0.0011	-0.84	2.01	vdW
**2**	O57…C2 (H_2O…AC)	3.13	0.0091	0.031	0.0010	-0.85	1.33	vdW
**3**	O57…Cl51 (H_2O…P)	3.06	0.0105	0.042	0.0016	-0.83	0.41	vdW
**4**	O57…Cl53 (H_2O…P)	3.14	0.0103	0.038	0.0012	-0.85	0.60	vdW
**5**	O57…C40 (H_2O…P)	2.50	0.0195	0.080	0.0022	-0.87	0.22	vdW
**6**	Cl53…C10 (P…AC)	3.23	0.0088	0.031	0.0016	-0.75	9.20	vdW
**7**	Cl42…C16 (P…AC)	3.41	0.0071	0.024	0.0012	-0.75	2.72	vdW

[1]Atoms interacting (above) and molecules interacting (below). AC: activated carbon; P: pesticide; H_2O: water molecule.
[2]The interactions were classified according to Nakanishi's criteria [49,50].
[3]vdW: van der Waals dispersive interaction; HB: hydrogen bond; HB-w: hydrogen bond weak; CT: charge transfer.
